# Effects of deep brain stimulation frequency on eye movements and cognitive control

**DOI:** 10.1038/s41531-023-00470-8

**Published:** 2023-03-31

**Authors:** André Zacharia, Diego Kaski, Walid Bouthour, Viswas Dayal, Matthieu Bereau, Philipp Mahlknecht, Dejan Georgiev, Julie Péron, Tom Foltynie, Ludvic Zrinzo, Marjan Jahanshahi, John Rothwell, Patricia Limousin

**Affiliations:** 1 Department of Clinical and Movement Neurosciences, UCL Queen Square Institute of Neurology, The National Hospital for Neurology and Neurosurgery, London, UK; 2 Department of Neurology, Geneva University Hospitals, Geneva, Switzerland; 3 Department of Neurology, Clinique Bernoise Montana, Crans-, Montana, Switzerland; 4 Service of Neurology, Department of Clinical Neurosciences, Lausanne University Hospital and University of Lausanne, Lausanne, Switzerland; 5 Department of Neurology, Besançon University Hospital, Besançon, France; 6 Department of Neurology, Innsbruck Medical University, Innsbruck, Austria; 7 Department of Neurology, University Medical Centre, Ljubljana, Slovenia

**Keywords:** Parkinson's disease, Parkinson's disease

## Abstract

Deep brain stimulation (DBS) of the subthalamic nucleus (STN) is an effective treatment for Parkinson’s disease (PD). Varying the frequency DBS has differential effects on axial and distal limb functions, suggesting differing modulation of relevant pathways. The STN is also a critical node in oculomotor and associative networks, but the effect of stimulation frequency on these networks remains unknown. This study aimed to investigate the effects of 80 hz vs. 130 Hz frequency STN-DBS on eye movements and executive control. Twenty-one STN-DBS PD patients receiving 130 Hz vs. 80 Hz stimulation were compared to a healthy control group (*n* = 16). All participants were tested twice in a double-blind manner. We examined prosaccades (latency and gain) and antisaccades (latency of correct and incorrect antisaccades, error rate and gain of the correct antisaccades). Executive function was tested with the Stroop task. The motor condition was assessed using Unified Parkinson’s Disease Rating Scale part III. The antisaccadic error rate was higher in patients (*p* = 0.0113), more so in patients on 80 Hz compared to 130 Hz (*p* = 0.001) stimulation. The differences between patients and controls and between frequencies for all other eye-movements or cognitive measures were not statistically significant. We show that 80 Hz STN-DBS in PD reduces the ability to maintain stable fixation but does not alter inhibition, resulting in a higher antisaccade error rate presumably due to less efficient fixation, without altering the motor state. This provides a wider range of stimulation parameters that can reduce specific DBS-related effects without affecting motor outcomes.

## Introduction

Deep brain stimulation of the subthalamic nucleus (STN-DBS) is an established treatment option for advanced Parkinson’s disease (PD)^1^. Stimulation at 130 Hz has been used since early studies because of remarkable short- and long-term results for core motor symptoms^1–4^. Some studies have subsequently explored the effects of lower stimulation frequencies on motor function either by changing the frequency acutely or applying it over longer period of time: very low frequency (10–30 Hz) worsens motor control but improves verbal fluency in PD^5^, low frequency (60–80 Hz) improves freezing of gait^6,7^ and speech^8,9^. In contrast, no effect on dexterity was observed with an acute change in frequency in the range of 40–160 Hz^10,11^. One study compared the long-term efficacy of 60 Hz vs. 130 Hz in “stand-walk-sit-test” and freezing episodes; this showed a sustained benefit with 60 Hz for up to 14.5 months^12^.

Given the inevitable overlap between STN-DBS and eye movement control networks, DBS also affects oculomotor function^13,14^. Because eye movement pathways are well-characterised in animals and humans, examining the effects of DBS on eye movements may offer insights into the mechanisms by which DBS operates. STN-DBS micro-recordings in PD patients have confirmed that neurons involved in saccade generation are located in the ventral part of STN^15^. Contraversive saccades can be generated when one STN is stimulated at a time^16^, suggesting a lateralized neural organisation. It is hypothesised that STN-DBS reduces the degree of inhibition exerted by the substantia nigra pars reticulata on the superior colliculus (SC)^17^ that is responsible for saccade initiation. Several studies have examined the effects STN-DBS on eye movements on and off dopaminergic medication^18^. Compared to treatment off stimulation, STN-DBS reduces pro-saccadic latency and improves the gain for visually-guided saccades^19–21^.

The basal ganglia oscillatory model of ocular function^21–24^ best explains the effects of STN-DBS effects on eye movements: beta-band oscillations are increased in PD patients, and because beta band desynchronization is required to initiate a motor command, motor thresholds are increased. STN-DBS thus decreases pathological oscillations and facilitates motor control, while stabilizing activity within SC, and restoring inhibitory saccadic control^25^, resulting in decreased latency and improved fixation.

Antisaccades (looking in the opposite direction to a suddenly appearing target) are of particular interest to PD as a measure of disinhibition^26^. Antisaccades are mediated by the frontal eye field (FEF) and SC^27,28^, with additional top-down influence from the supplementary eye fields (SEF) and dorsolateral prefrontal cortex (DLPFC)^27^, adding further cognitive layers to the final oculomotor execution and providing insights into executive function. STN-DBS has been shown to improve antisaccade gain^20,29^ and latency^21^ in PD. The number of errors during antisaccades was greater on high frequency STN-DBS in some^20^ but remained unchanged^21,30^ in other studies. These data suggest that STN overactivity can be altered by DBS to facilitate eye movements overall.

Executive processes are impaired in PD, with deficits in planning (initiation, maintenance, and monitoring) and attention that interfere with goal-directed behaviour^31^. Both the antisaccades and the Stroop task^32^ require inhibitory control of prepotent responses for correct performance^33^. Such tasks are altered in PD^31^ and may be worsened by STN-DBS^34–37^, with more errors in the Stroop interference task on vs. off DBS^38^ and longer reaction times in pre vs. post DBS at 130 Hz^39^.

Although a few studies have examined the effect of STN-DBS on eye movements and cognition, there are limited data on how different DBS frequencies may modulate specific eye movements and executive function. Given the overlap between the neural circuits for gait and eye movements^40^, and the fact that lower frequencies (60–80 Hz) were shown to improve gait, we sought to compare the effect of 80 Hz vs. 130 Hz STN-DBS on eye movements. Moreover, 80 Hz frequency is routinely used in our centre to try to improve speech and gait problems following DBS. We hypothesized that visually guided saccades might respond similarly to the appendicular motor system at different STN-DBS stimulation frequencies, i.e., reduced saccadic latency (reaction time) with high frequency stimulation. We also predicted that more complex eye movements such as antisaccades might be more affected by 130 Hz STN-DBS than by 80 Hz (decreased saccade latencies and increased errors). Similar effects of STN-DBS frequency would also be expected in the interference subtasks of the Stroop task. These predictions are based on previous evidence showing that tasks with higher cognitive load are affected by high frequency DBS^41–43^ but better accomplished when the DBS frequency is lowered^10^.

## Results

One patient was excluded from the motor state and eye movement analysis since he did not tolerate the change in frequency from his usual 60 Hz to 130 Hz and 80 Hz due to worsening gait impairment. Therefore, data from 20 PD patients and 16 HC were subjected to further analysis.

### Motor assessment

The UPDRS total motor score and subscores for bradykinesia, rigidity, tremor and axial subscore were not significantly different between the two frequencies, all *p* > 0.2201 (Table 1).

**Table 1:** The means and standard deviations (SD, in parentheses) of the prosaccade, antisaccade, and Stroop task measurements for Parkinson’s disease patients (PD) at 130 and 80 Hz stimulation frequencies and for the healthy controls (HC) on the two days and the results of the generalized mixed-effects model (F) for repeated measures are shown.

	PD		HC		Main effect	Interaction	Main effect	Main effect	Interaction
	130 Hz	80 Hz	1st Time	2nd Time	Order	Group × Order	Group	Frequency/Time	Group × Frequency/Time
	mean (SD)	mean (SD)	mean (SD)	mean (SD)	*p*-value (F)	*p*-value (F)	*p*-value (F)	*p*-value (F)	*p*-value (F)
	**Prosaccade task (n** **=** **20)**								
Latency *(ms)*	170.57 (39.94)	160.67 (34.76)	136.65 (23.71)	151.81 (39.28)	0.1190 (4.95)	0.5830 (0.33)	0.1190 (4.95)	0.1191 (3.95)	0.5831 (0.31)
Gain	0.89 (0.08)	0.93 (0.06)	0.92 (0.04)	0.92 (0.04)	0.8474 (0.24)	0.8474 (0.12)	0.1425 (3.81)	0.8396 (0.51)	0.1425 (4.4)
	**Antisaccade task (n** **=** **20)**								
Latency Incorrect *(ms)*	171.37 (36.29)	177.44 (40.2)	155.62 (52.14)	164.43 (36.75)	0.8640 (0.11)	0.8640 (0.91)	0.9908 (0)	0.8640 (1.91)	0.8640 (0.41)
Latency Correct *(ms)*	317.35 (88.22)	366.94 (130.45)	284.38 (57.09)	281.67 (71.24)	0.1765 (2.17)	0.1765 (2.44)	0.1765 (2.54)	0.1341 (4.67)	0.1765 (2.26)
Error Rate	44.41 (26.29)	59.52 (23.2)	23.5 (15.53)	21.8 (14.59)	0.8280 (0.25)	0.8280 (0.14)	**0.0113** **(9.06)**	**0.001** **(16.72)**	**0.0113** **(8.6)**
Gain Correct	0.9 (0.2)	0.95 (0.3)	0.95 (0.3)	1.01 (0.31)	0.8059 (2.63)	0.8194 (0.05)	0.8194 (0.08)	0.8093 (0.5)	0.8093 (1.14)
	**Stroop task (n** **=** **18)**								
Inhibition task *(s)*	68.83 (23.23)	71.44 (21.78)	66 (19.47)	58.25 (13.31)	**0.0076** (8.38)	0.0939 (4.02)	0.0939 (4.32)	0.3075 (1.57)	0.3624 (1.06)
Inhibition task *(number of errors)*	1.89 (3.76)	1.94 (3.89)	0.38 (1.5)	0.38 (1.02)	1 (0.51)	1 (0.94)	1 (0.01)	0.7371 (2.78)	1 (0.01)
Switching task *(S)*	94.22 (41.04)	87.28 (39.32)	71.25 (19.93)	67.94 (27.38)	0.1316 (2.8)	0.8683 (0.03)	0.5738 (0.48)	0.1316 (3.55)	0.1316 (3.86)
Switching task *(number of errors)*	2.67 (3.41)	1.56 (2.89)	1.38 (2.6)	1 (2)	0.3161 (2.12)	0.7859 (0.08)	0.443 (1.32)	0.443 (1.04)	0.0947 (5.37)
Stroop Effect 1 *(s)*	32.83 (18.86)	35.06 (15.52)	30.06 (20)	23.25 (10.21)	0.0619 (5.41)	0.1882 (3.61)	0.1882 (2.74)	0.219 (2.11)	0.4742 (0.71)
Stroop Effect 2 *(s)*	30.44 (32.93)	23.06 (34.6)	0.81 (15.15)	8.75 (23.33)	0.1351 (3.6)	0.8688 (0.03)	0.3954 (1.2)	0.137 (3.31)	0.1370 (3.76)
Stroop Effect 3 *(s)*	25.39 (27.24)	15.83 (26.59)	5.25 (15.77)	9.69 (20.1)	**0.0103** (7.83)	0.5189 (0.63)	0.1518 (3.15)	0.1518 (3.6)	0.5208 (0.42)
	**UPDRS-III**	**(n** **=** **20)**							
UPDRS III Total Score	19.2 (9.1)	16.9 (10.5)							0.2300 (*t* = 1.51)
Bradykinesia	8.7 (5.1)	7.7 (5.6)							0.4000 (*t* = 0.9)
Rigidity	3.6 (3.4)	3.2 (3.2)							0.5000 (*Z* = 72)
Axial	2.5 (2.2)	2.2 (2.1)							0.3000 (*Z* = 60)
Tremor	1.3 (1.4)	1.5 (1.8)							0.5000 (*Z* = 40)

To test the repetition effect, repeated measures ANOVA (factors Group and Order) were performed. To test the frequency change effect, mixed measures ANOVA (factors Group and Frequency/Time) were performed. Wilcoxon test and *t* tests comparing the Unified Parkinson’s Disease Rating Scale part III (UPDRS-III) and sub-scores between both frequencies were also performed. Please see the manuscript for details. Significant results are shown in bold.

### Prosaccades

No repetition effect was observed for saccade gain or latency in either group (*p* > 0.1180). There were no significant differences in latency (*p* = 0.1190) and gain (0.1425) between PD and HC. Similarly, there were no significant differences in neither latency (*p* = 0.1191), nor gain (*p* = 0.8396) at the different stimulation frequencies. There was no significant interaction effect of Group×Frequency/Time for gain and latency (*p* > 0.1424).

### Antisaccades

As with the prosaccades, no repetition effect was observed in the latencies for correct and incorrect antisaccades, error rate, or gain of correct antisaccades in either group (all *p* > 0.1764). The antisaccadic error rate was higher in patients (*p* = 0.0113), and it was higher in patients on 80 Hz compared to 130 Hz (*p* = 0.001). The interaction effect of Group×Frequency/Time for error rate was also significant (*p* = 0.0113). There was no significant main or interaction effect of Group and Frequency/Order for latency of incorrect, latency of correct antisaccades and gain of the correct antisaccades (all *p* > 0.1340), Table 1.

For both prosaccades and antisaccades, we did not observe any decline in task performance over time, across either stimulation parameter group, suggesting no effects of fatigue or task duration.

### Stroop task

Of the 21 patients, three patients were excluded from the analysis of the Stroop task, one patient because of poor performance on the control task, the second was illiterate, and the third patient did not tolerate the frequency change. Therefore, 18 patients performed the Stroop task.

A learning effect was observed in the time taken to complete the test both for the Inhibition task (*p* = 0.0076) and for the Stroop effect 3 (*p* = 0.0103). However, there were no significant effect of neither group nor frequency of stimulation on any of the measures of the Stroop task (all ps > 0.0946), Table 1.

### Correlation between antisaccade errors and latency

To test the correlation between antisaccade latency and error rate, we first confirmed a tight correlation between the incorrect and correct response latencies for both the patients and the control group (*r* = 0.5, *p* < 0.001, for both groups). We further explored the correlation between the antisaccade error rate and the grouped antisaccade latency (for both incorrect and correct saccades) for both groups and both stimulation frequencies. No correlation was found in the control group (*p* > 0.05). However, the error rate for patients negatively correlated with saccade latency at both 130 Hz (*r* = −0.62, *p* = 0.0072) and 80 Hz (*r* = −0.56, *p* = 0.0201) (more errors associated with shorter saccade latency). Linear regression showed that 34% of the error rate variance was explained by saccade latency when patients were stimulated at 130 Hz and 27% when they were stimulated at 80 Hz.

### Correlation between the Stroop effects and antisaccadic error rate

No significant correlations were found for the Stroop effect 1, 2 and 3 for the stimulation at 130 Hz, at 80 Hz and in the control group (all ps > 0.1999).

## Discussion

We investigated the influence of STN-DBS stimulation at 130 Hz and 80 Hz on oculomotor saccade function and cognitive control in PD patients compared to an age-matched control group. We demonstrated that for some eye movements, performance was dependent on stimulation frequency; thus the antisaccade error rate was higher at 80 Hz compared with 130 Hz stimulation. Simply, patients stimulated at a 80 Hz perform less well in an antisaccade task. Interestingly, whilst stimulation at 80 Hz resulted in more antisaccade errors, the number of errors during the Stroop inhibition/switching subtest remained unchanged. These results suggest a differential modulation of oculomotor and cortical executive cognitive control by STN-DBS.

Contrary to other studies showing that STN-DBS in PD decreases pro-saccadic latency – the time required to initiate a saccade - and increases velocity and amplitude of visually-guided saccades^21,23,44–46^, we did not find an effect of stimulation on prosaccadic latency. Other studies report a positive effect of STN-DBS on visually guided and voluntary saccades^21,25,46,47^, whereas in some studies, specific parameters (e.g., latency) are improved only for visually guided saccades and other parameters (gain of the first saccade) are improved only for voluntary saccades^22,24,29,30,44,48,49^. In addition, one might argue that the effect of STN-DBS could be mediated by direct stimulation of the oculomotor nerve passing medially to the STN. We think this is not the case since this side effect is usually permanent and that our patients did not complain of diplopia^50^

While it is generally accepted that STN-DBS increases antisaccade latency in PD patients^51,52^, there is a wide variability in reported STN-DBS effects on antisaccade error rate. We did not find an effect of stimulation on antisaccade latency. We found higher antisaccade error rates in patients stimulated at 80 Hz STN-DBS, which could be due either to a direct effect on facilitating eye movements, or to a more general impairment of executive control and, in particular, to a loss of inhibitory control leading to disinhibition and higher impulsivity. The mechanism underlying this finding could may be due to the fact that SC is disinhibited by STN-DBS^17,21^ and, according to our results, even more so at 80 Hz than 130 Hz (better prosaccade accuracy but more errors in antisaccades at 80 Hz). Cortical areas involved in saccade generation during the antisaccade task include the FEF and the DLPFC^28,53,54^; lesions of the former lead to increased latencies but not errors, whereas lesions of to the latter lead to increased saccade errors without altering the latency^27^.

The fact that antisaccade error rate was greater in patients receiving STN-DBS compared to control subjects would argue against a direct effect of STN on SC (since only latencies should be increased). Instead, it has been suggested that STN-DBS interferes with the interloop transfer: transfer between the oculomotor loop and the prefrontal oculomotor loop at the level of the striatum. The prefrontal oculomotor loop involves the DLPFC and the basal ganglia oculomotor centres (STN, striatum, and ventrolateral thalamus). It has been proposed that this loop is responsible for the control of complex eye movements (including the initiation of voluntary prosaccades and also the inhibition of prosaccades in the antisaccade task) by acting on the oculomotor loop (which originates in the FEF/SEF, goes to the STN and striatum, then to the oculomotor thalamus and back to the FEF/SEF), as part of a double loop hypothesis^54^. If antisaccade error rate is indeed related to abnormalities of fixation, one might expect higher frequency of saccadic oscillations interfering with fixation in STN DBS (e.g., square wave jerks). Whilst we did not observe clear differences in the frequency of square wave jerks in the higher DBS stimulation frequency condition on visual inspection of the data, these were not objectively assessed or quantified, a feature that future studies may wish to explore.

We hypothesized that antisaccades, being a more cognitively demanding task^55^, would be more affected by 130 Hz than by 80 Hz stimulation. We did not find effect of stimulation on the Stroop task, but contrary to prediction an aggravation of the antisaccade error rate. The reason for this discrepancy could be that the antisaccade task not only involves working memory but also requires an efficient fixation system capable of maintaining a saccade despite the gap. This is consistent with a study that showed no correlation between correct antisaccades and the Stroop effect^56^. These results highlight that while both the antisaccade and Stroop task require inhibitory control, their modulation by STN-DBS may be different. It has been shown that STN-DBS can modify the speed-accuracy tradeoffs in favour of speed^42,57^, and this would account for reduced saccadic latencies with increased saccadic errors. Jahanshahi et al. have shown that the acute effects of 130 Hz STN-DBS on different executive tests are not uniform; for example STN-DBS did not affect verbal fluency, but the results on the Trail-Making test and Wisconsin Card Sorting were improved^38^.Whether this is due to differences in the anatomical, electrophysiological, and/or functional properties of the affected cortical and subcortical networks in different cognitive domains remains to be elucidated^58^. Interestingly, we report that our control group exhibited a learning effect when repeating the Stroop task for the time completion, whereas this was not the case in the patients. While such a learning effect might be expected in the control group, its absence probably reflects executive dysfunction in the patient group^59^.

As a limitation to our findings, we acknowledge that we might have reached a ceiling effect of the two different stimulation conditions since the patients were not tested in ‘off’ stimulation. However, (although this was technically possible) this was decided in the study design stage to avoid an additional burden on patients. In addition, this study was designed to assess the chronic effect of both frequencies rather than acute effects, specifically attempting to avoid compensatory mechanisms that may alter oculomotor or cognitive function. We cannot fully exclude however that such compensatory mechanism may have influenced the results.

From a clinical perspective, our data show that lowering the stimulation frequency from 130 Hz to 80 Hz can modify eye movement performance without affecting motor symptoms, thus providing a wider range of stimulation parameters that can reduce specific DBS-related side effects without compromising motor outcomes.

Whether oculomotor function correlates with the location of the DBS lead location would be interesting to study in the future, but may require a greater number of participants.

## Methods

### Participants

We enrolled 21 consecutive PD patients diagnosed by the Brain Bank Criteria with STN-DBS and 16 age-matched healthy controls (HC). Patients had received chronic STN-DBS for more than six months at either the National Hospital for Neurology and Neurosurgery London (13 patients) and the University Hospital of Geneva (8 patients) and were recruited consecutively from the respective outpatient clinics. Patient selection required willingness to adhere to this study. Patients were implanted bilaterally with standard DBS electrodes (3389, Medtronic) and were on their usual DBS settings at baseline. The surgical procedures performed followed the usual routine for each respective center^60,61^. Exclusion criteria were the presence of a visual and spatial neglect or blepharospasm detected during a routine neurological examination. Only patients with corrected refractory visual abnormalities were included in the study. To assess the effect of stimulation alone, patients were tested off medication after overnight withdrawal of dopaminergic medication. Stimulation parameters were changed to the two stimulation frequencies: 80 Hz (less commonly used, usually to treat the long-term gait disturbances after DBS) and 130 Hz (the most commonly used stimulation frequency). Any potential confounder of pre-study stimulation frequency (note that some patients were receiving a lower [80 Hz] frequency prior to the study) was counterbalanced by the randomization process. Both the examiner and the patient were blinded to the stimulation setting. All participants gave informed written consent. Control subjects were either the patients’ spouses or recruited from a list of volunteers. They had no history of psychiatric or neurological conditions and were matched for age, sex, education, and global cognitive performance on the Mini-Mental State Examination (MMSE)^62^. The mean score of the Frontal Assessment Battery (FAB)^63^ was lower in PD (Table 2). The study was conducted in accordance with the Declaration of Helsinki and approved by both local ethics committees (Health Research Authority NRES Commitee London 13/LO/1255, Commision cantonale d’éthique de la recherche de Genève, projet 15–300).

**Table 2:** Demographic and clinical data of patients and control subjects.

	Control subjects	PD patients	*p*-value
*N*	16	21	
Age	66.06 (5.37)	63.71 (9.16)	0.8
Gender (M|F)	10 | 6	15 | 6	0.2
Handedness (R|L)	16 | 0	19 | 2	0.2
MMSE	28.7 (1.4)	28.8 (1.2)	0.9
Education level	1.68 (0.74)	1.75 (0.84)	0.8
FAB	17.06 (1.34)	15.1 (2.95)	**0.04**
UPDRS part III total		14.52 (6.7)	
Time since DBS (years)		3.87 (1.5)	
LEDD (mg)		560.81 (298.5)	
Frequency STN Right (Hz)		112.86 (24.73)	
Frequency STN Left (Hz)		115.71 (29.08)	
Voltage STN Right (V)		3.03 (0.73)	
Voltage STN Left (V)		2.85 (0.72)	
Pulse width (µS)		60 (0)	
Impedance Right STN (Ohms)		1190 (238.64)	
Impedance Left STN (Ohms)		1071.4 (199.3)	

Mean parameters of deep brain stimulation. Standard deviations of the mean are given in parentheses. *FAB* Frontal Assessment Battery, *LEDD* Levodopa Equivalence Daily Dose, *MMSE* Mini Mental State Examination, *STN* Subthalamic Nucleus, *UPDRS III* Unified Parkinson’s Disease Rating Scale part III at baseline, *M* men, *F* women, *R* right-handed, *L* left-handed. Statistically significant differences are marked in bold.

### Baseline visit

A baseline visit (1 to 2 weeks before testing the participants) was organized to administer the MMSE and the FAB to exclude patients with major cognitive impairment (MMSE<24) and dysexecutive syndrome (FAB<12)^62,63^ and patients meeting the other exclusion criteria.

### Experimental setup

The study was double-blind. Settings were randomized and matched by a neurologist who did not participate in the assessments. Patients and controls completed two separate testing sessions on two consecutive days. Patients were tested after a 12 h overnight withdrawal from dopaminergic medication at 1) 130 Hz, or 2) 80 Hz stimulation. We attempted to isolate frequency of stimulation effects from total energy delivered by adjusting the voltage according to the total energy being delivered, as per Koss et al.^64^. At least 20 h at each frequency prior to testing was mandatory to ensure adequate time under a given stimulation^65^. The order of stimulation frequency was randomized between patients at the end of the baseline assessment using a random number generator. Patients did not undergo formal follow-up as part of the study, but any adjustments to the DBS parameters were carried out on clinical grounds, as required.

### Motor assessment

Motor assessment was performed using Unified Parkinson’s Disease Rating Scale (UPDRS) part III^66^. A *bradykinesia* subscore was calculated by adding the scores for items 23, 24, 25, and 26. A *rigidity* subscore was calculated by adding the five scores for item 22 (neck, left and right upper extremity and left and right lower extremity). An *axial* subscore was calculated by adding the scores for items 27, 28, 29 and 30. Finally, a *tremor* subscore was calculated by adding the scores for items 20 and 21 from the UPDRS part III.

### Eye movements recording

**Fig. 1:**
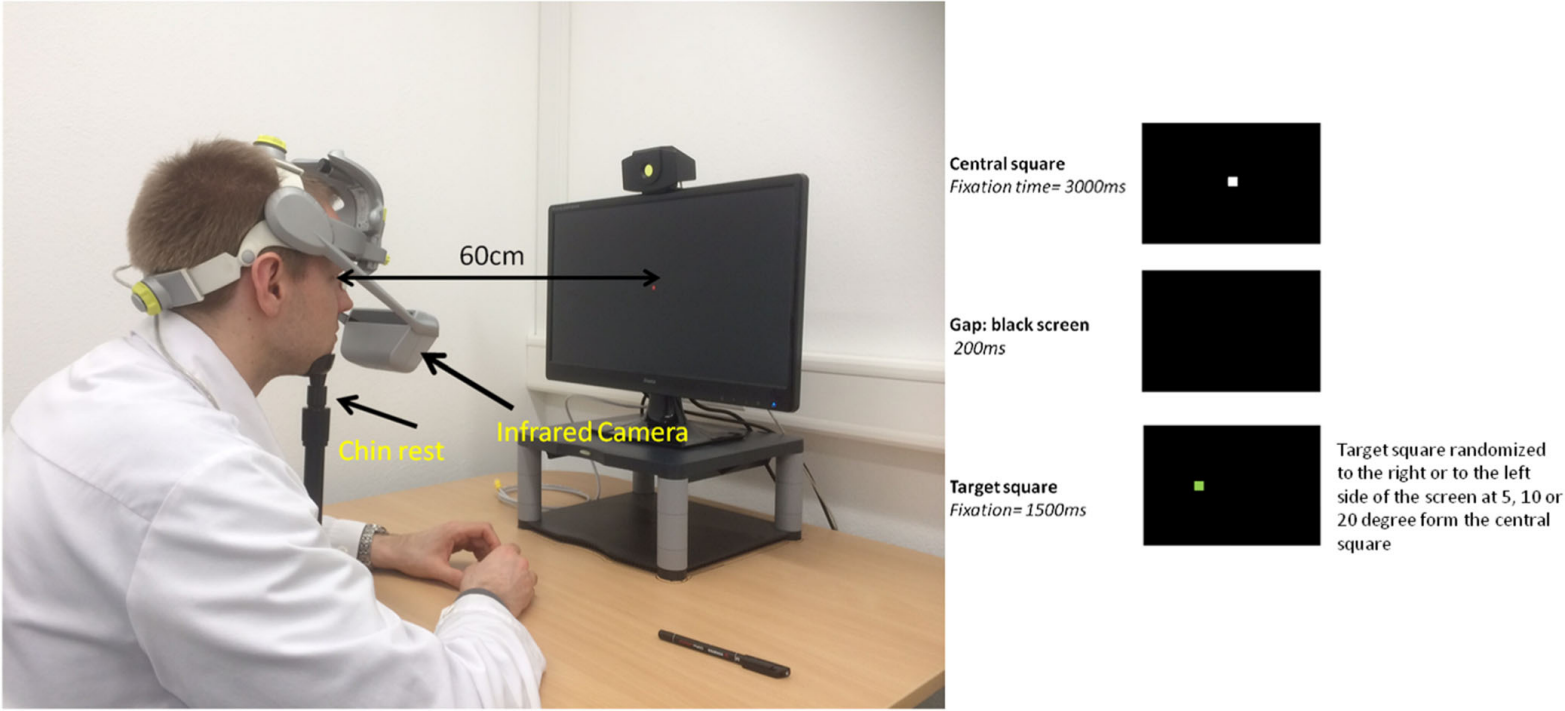
Eye-tracker montage and sequence of events during a given trial. The sequence of events was identical on the prosaccade and antisaccade trials. For prosaccades, the instruction was, “Look as fast as you can to the target square when it appears and come back to the central point.” On antisaccade trials, subjects were instructed, “When the green square appears, please look as quickly and accurately as possible to the mirror-image location in the opposite direction. Try to make an eye movement toward the target.” The individual on this figure is a co-author (M, B). He has given his consent for the publication of his image.

We used a head-mounted eye tracker system (Mobile EBTH, e(ye)BRAIN, www.eyebrain.com) with an infrared camera to record eye movements while simultaneously recording head movements. Participants wore a padded helmet that kept the camera fixed in front of their eyes without obscuring the visual field. The recording frequency was 300 Hz. A chin rest was used to reduce head movements (Fig. 1). The system was used in a room without windows to ensure that the same amount of light is present during each session. The measurements were performed in darkness. To avoid fatigue, breaks were provided throughout the testing protocol. In addition, we ensured each session was as short as possible.

### Prosaccades – gap condition

We recorded 24 trials per condition. 1 or 2 practice trials were conducted to ensure task comprehension. A trial was set up as follows: 1) A white central fixation square appeared on a black screen for 3000 ms; 2) Then, a black screen representing the gap appeared for 200 ms, then; 3) A green target square was randomly assigned either to the right or left of the central fixation square for 1500 ms, at visual angles of 5°, 10°, and 20° (Fig. 1). The instructions were as follows: “Look at the green squares when they appear, as quickly and accurately as possible. Please try to make an eye movement toward the target.”

Saccades were automatically detected by an algorithm in the software MeyeAnalysis, software that came with the eye tracker. Saccades were defined as follows: an abrupt eye movement reaching a velocity threshold of >30°/s and an amplitude of 2–40°. Only the first saccade that occurred after the onset of the peripheral stimuli (the cue) was analysed. The latency for saccade initiation was >80 ms (shorter latencies were considered anticipatory). Detected saccades were visually inspected and discarded if a blink occurred at the beginning of the saccade or if gaze did not return to the baseline before the next cue appeared on the screen. Gain, latency, and amplitude were calculated. *Gain* was defined as the ratio between the actual saccade divided by the maximum amplitude of the saccade to the respective target. *Saccade latency* was defined as the time between the cue and the onset of the saccade. *Saccade amplitude* was defined as the distance (in degrees) between the saccade start and the first landing point.

### Antisaccades

**Fig. 2:**
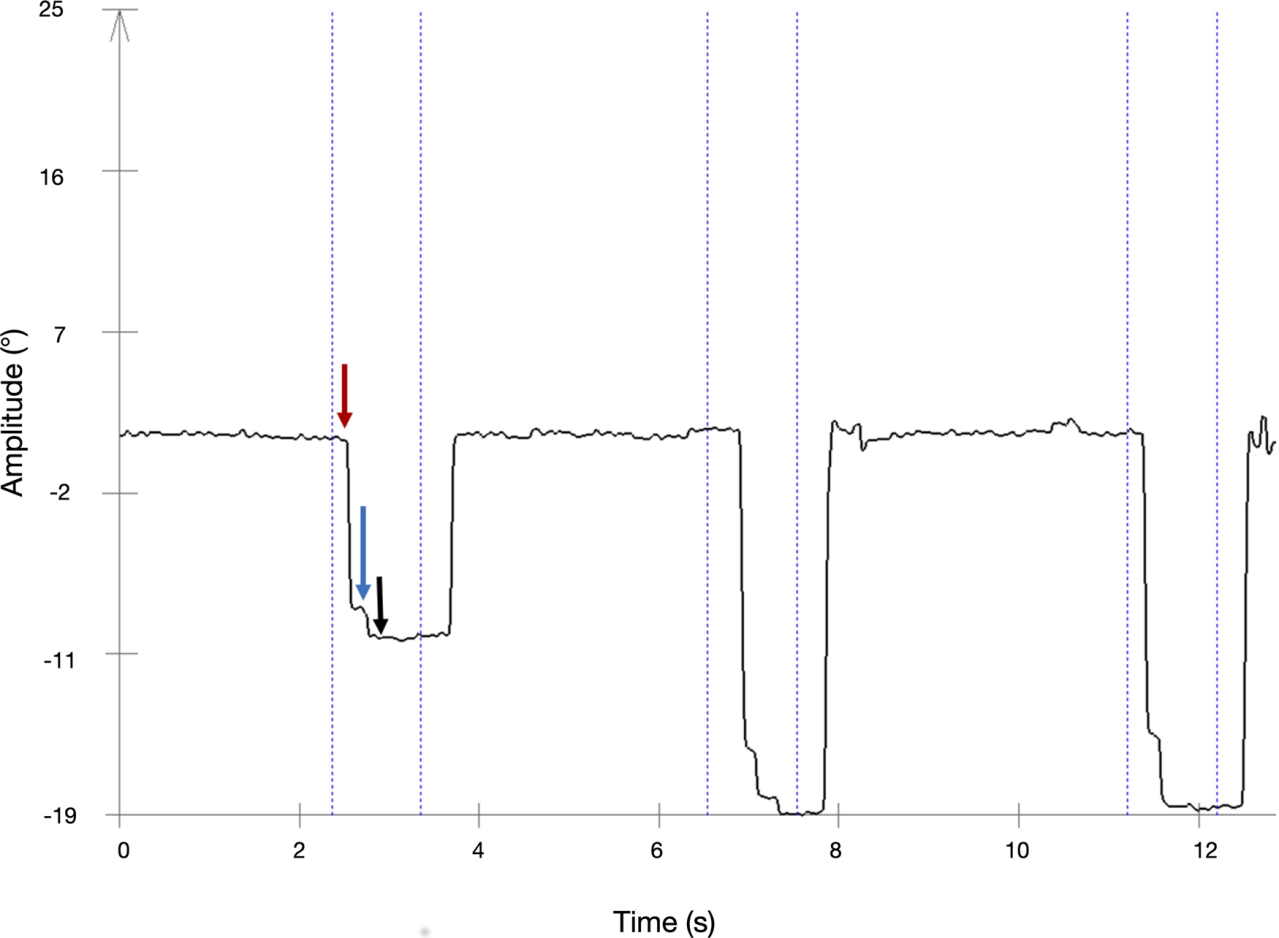
Oculographic recording of the left eye of a representative patient with PD, undergoing STN-DBS stimulation at 130 Hz. Traces represent horizontal eye movements (a downward deflection corresponds to a leftward eye movement). The onset of a saccade (vertical red arrow) was automatically detected using the eye-tracker software, but all traces were visually inspected for consistency and accuracy. The blue arrow indicates the first ‘landing point’ of the saccade trajectory (hypometry in this example), and the black arrow indicates the end of the saccade. Saccade latency was measured as the time between stimulus onset (dotted blue line) and saccade onset (red arrow). Saccade gain was calculated as the ratio between the amplitude of the first saccadic “landing point” (approximately 7.5 degrees, blue arrow) and the maximum saccadic amplitude (approximately 10 degrees, for the saccade in this example).

The same gap paradigm was used for the antisaccades task. However, participants were asked to look in the opposite direction in which the square appeared. The instructions were as follows: “When the green square appears, please look as quickly and accurately as possible in the mirror-image position in the opposite direction. Try to make one eye movement toward the target.” This task was performed in a block of 24 trials per condition. We calculated the *number of incorrect prosaccades*, *saccade latencies* (the time between the stimulus appearance and the onset of the saccade) for both correctly performed antisaccades and the incorrect saccades, and *gain* (the ratio between the saccade amplitude and stimulus amplitude) for correct antisaccades (Fig. 2).

### The Stroop task

The Stroop colour word interference task from the Delis–Kaplan battery^67^ includes the following four subtasks: 1) Control naming task: In this task the patients is asked to name as quickly as possible the colour of individual rectangles that appear on the screen; 2) Reading task: In this task the patient is asked to read as quickly as possible the colour words red, blue, and green printed in black ink 3) Inhibition task: In this task the patient is asked to name as quickly as possible the colour of the colour words red, green, blue - and do not read the word itself. In this task the words are printed in an inconsistent colour ink (e.g., the word “red” is printed in green ink); 4) Inhibition/switching task: In this task the patient is again presented with the words “red”, “green”, and “blue” in inconsistent colours of red, green, or blue ink. Half of these words are enclosed in boxes. As in the Inhibition task, the patient is asked to name the colour of ink in which each word is printed, but to read the word aloud when a word appears in a box as quickly as possible without making mistakes^68^ This task requires more working memory than the inhibition trial and captures the abilities to switch sets^68–70^.

Participants were instructed to complete each subtask as quickly as possible and to correct themselves if they made any errors. The total time taken to complete each subtask and the number of self-corrected and uncorrected trials were recorded. We also calculated Stroop effects, defined as the time difference between the Control naming task and the Inhibition task (Stroop effect 1), between the Control naming and Inhibition/switching task (Stroop effect 2), and between the sum of Control naming and Reading task and the Inhibition/Switching task (Stroop effect 3).

### Statistical analysis

The analysis was divided into three steps to evaluate: 1) The effect of task repetition; 2) The differences between HC and PD in task performance; 3) The effect of stimulation frequency. For each step, we checked the distribution assumptions and used a generalized mixed-effect regression model that takes into account missing data^71^. The Shapiro–Wilk test was used to check normality. We used the statistical package lme4^72^ for the R software^73^. Stimulation frequency was set as a fixed effect (130 Hz vs. 80 Hz). To test the effect of order of testing, repeated measures ANOVA were performed with a fixed factor of order (1st vs 2nd Time). To assess the effect of group and frequency, a mixed repeated measures model ANOVA was performed with the within-subject factor Group (HC and PD) and the within-subject factor Frequency/Time (80 vs. 130 Hz/1st vs. 2nd Time). Motor scores and subscores were compared using the *t*-test or Wilcoxon test depending on the normality of the distribution. Pearson’s r correlation coefficient and linear regression analysis was used for correlation analysis. The significance value was two-tailed set at *p* ≤ 0.05. False Discovery Rate^74^ was used to correct for multiple comparisons.

### Reporting summary

Further information on research design is available in the Nature Portfolio Reporting Summary linked to this article.

## Supplementary information

Reporting Summary

## Data Availability

Access to anonymised patient data is possible upon request to the corresponding author.

## Code availability

Access to R codes used for the statistical analysis is possible upon request to the corresponding author.
