# Supplementary material for: Effects of deep brain stimulation frequency on eye movements and cognitive control
Source: NPJ Parkinsons Dis. 2023 Mar 31;9:50. doi: 10.1038/s41531-023-00470-8 (PMC10066205; doi:10.1038/s41531-023-00470-8)
Supplement: Supplementary file 1 — Reporting Summary [file 41531_2023_470_MOESM1_ESM.pdf]

nature portfolio

DBPR
NPJPARKD-01449
Corresponding author(s): André ZACHARIA

Last updated by author(s): Oct 17, 2022

Reporting Summary

Nature Portfolio wishes to improve the reproducibility of the work that we publish. This form provides structure for consistency and transparency in reporting. For further information on Nature Portfolio policies, see our Editorial Policies and the Editorial Policy Checklist.

Statistics

For all statistical analyses, confirm that the following items are present in the figure legend, table legend, main text, or Methods section.

n/a	Confirmed	
☐	☒	The exact sample size (*n*) for each experimental group/condition, given as a discrete number and unit of measurement
☐	☒	A statement on whether measurements were taken from distinct samples or whether the same sample was measured repeatedly
☐	☒	The statistical test(s) used AND whether they are one- or two-sided<br>*Only common tests should be described solely by name; describe more complex techniques in the Methods section.*
☐	☒	A description of all covariates tested
☐	☒	A description of any assumptions or corrections, such as tests of normality and adjustment for multiple comparisons
☐	☒	A full description of the statistical parameters including central tendency (e.g. means) or other basic estimates (e.g. regression coefficient) AND variation (e.g. standard deviation) or associated estimates of uncertainty (e.g. confidence intervals)
☐	☒	For null hypothesis testing, the test statistic (e.g. *F*, *t*, *r*) with confidence intervals, effect sizes, degrees of freedom and *P* value noted<br>*Give P values as exact values whenever suitable.*
☒	☐	For Bayesian analysis, information on the choice of priors and Markov chain Monte Carlo settings
☒	☐	For hierarchical and complex designs, identification of the appropriate level for tests and full reporting of outcomes
☒	☐	Estimates of effect sizes (e.g. Cohen's *d*, Pearson's *r*), indicating how they were calculated

Our web collection on statistics for biologists contains articles on many of the points above.

Software and code

Policy information about availability of computer code

Data collection	MeyeAnalysis, excel
Data analysis	Pinheiro, J., Bates, D., DebRoy, S., Sarkar, D. & R Core Team. nlme: Linear and Nonlinear Mixed Effects Models. (2013).<br><br>R Core Team. R: A Language and Environment for Statistical Computing. (2013).

For manuscripts utilizing custom algorithms or software that are central to the research but not yet described in published literature, software must be made available to editors and reviewers. We strongly encourage code deposition in a community repository (e.g. GitHub). See the Nature Portfolio guidelines for submitting code & software for further information.

Data

Policy information about availability of data

All manuscripts must include a data availability statement. This statement should provide the following information, where applicable:

- Accession codes, unique identifiers, or web links for publicly available datasets
- A description of any restrictions on data availability
- For clinical datasets or third party data, please ensure that the statement adheres to our policy

The datasets generated during and/or analysed during the current study are available from the corresponding author on reasonable request.

Human research participants

Policy information about studies involving human research participants and Sex and Gender in Research.

Reporting on sex and gender	Sex was assigned based on self reporting
Population characteristics	see table 1, patient characteristics
Recruitment	Patients had received chronic STN-DBS for more than six months at either the National Hospital for Neurology and Neurosurgery London (13 patients) and the University Hospital of Geneva (8 patients) and were recruited consecutively from the respective outpatient clinics. Patient selection required willingness to adhere to this study.
Ethics oversight	NHS health research authority NRES commitee, Clinical and neurophysiological assessments of DBS frequency in Parkinson's disease, 13/LO/1255<br>Commission cantonale d'éthique de la recherche de Genève [CCER] projet 15-300

Note that full information on the approval of the study protocol must also be provided in the manuscript.

Field-specific reporting

Please select the one below that is the best fit for your research. If you are not sure, read the appropriate sections before making your selection.

☒ Life sciences ☐ Behavioural & social sciences ☐ Ecological, evolutionary & environmental sciences

For a reference copy of the document with all sections, see nature.com/documents/nr-reporting-summary-flat.pdf

Life sciences study design

All studies must disclose on these points even when the disclosure is negative.

Sample size	Sample size was based on the number of subjects recruited according to similar studies in the same topic.
Data exclusions	Exclusion criteria were the presence of a visual and spatial neglect or blepharospasm detected during a routine neurological examination.<br>One patient was excluded from the motor state and eye movement analysis since he did not tolerate the change in frequency from his usual 60Hz to 130Hz and 80Hz due to worsening gait impairment.<br>Three patients were excluded from the analysis of the Stroop task, one patient because of poor performance on the control task, the second was illiterate, and the third patient did not tolerate the frequency change
Replication	We tried to explain in the methods the setting the most thoroughly possible in order to make this study replicable. In addition no patients were asked to repaet any of the tasks of the sake of reproducibility as customary in this type of research.
Randomization	The order of stimulation frequency was randomized between patients at the end of the baseline assessment using a random number generator
Blinding	As this study was meant exploratory, we made sure the the patients and the investigators did not know what setting was chosen. the DBS battery setting were modified by a neurologist not involved in the data analysis.

Reporting for specific materials, systems and methods

We require information from authors about some types of materials, experimental systems and methods used in many studies. Here, indicate whether each material, system or method listed is relevant to your study. If you are not sure if a list item applies to your research, read the appropriate section before selecting a response.

Materials & experimental systems

n/a	Involved in the study	
☒	☐	Antibodies
☒	☐	Eukaryotic cell lines
☒	☐	Palaeontology and archaeology
☒	☐	Animals and other organisms
☒	☐	Clinical data
☒	☐	Dual use research of concern

Methods

n/a	Involved in the study	
☒	☐	ChIP-seq
☒	☐	Flow cytometry
☒	☐	MRI-based neuroimaging
